# Bilateral Occlusion Reduces the Ocular Deviation in Intermittent Exotropia

**DOI:** 10.1167/iovs.62.1.6

**Published:** 2021-01-05

**Authors:** John R. Economides, Daniel L. Adams, Jonathan C. Horton

**Affiliations:** 1 Department of Ophthalmology, Program in Neuroscience, University of California, San Francisco, San Francisco, California, United States

**Keywords:** strabismus, intermittent exotropia, eye movements

## Abstract

**Purpose:**

The most common form of strabismus, intermittent exotropia, is thought to become manifest when the drive to fuse is overcome by excessive divergent muscle tone. This principle is tested by examining the alignment of the eyes in the absence of vision. We compare the ocular deviation in patients with intermittent exotropia under conditions of monocular versus binocular occlusion.

**Methods:**

This prospective study of a patient cohort referred to our laboratory enrolled 18 patients with typical findings of well-controlled intermittent exotropia. Eye positions were recorded with video eye trackers while patients looked at a fixation spot at a distance of 57 cm. One eye was occluded, and the resulting ocular deviation was measured. Both eyes were then occluded, and the ocular deviation was re-measured.

**Results:**

The majority of patients (11/18) had a smaller deviation when both eyes were covered. Occlusion of one eye resulted in a mean exotropia of 13.5° ± 4.7°. Occlusion of both eyes reduced the mean exotropia to 6.0° ± 6.5° (paired *t*-test, *P* < 0.001), corresponding to a 56% reduction in the ocular deviation. This reduction persisted during prolonged bilateral occlusion but reversed as soon as vision was restored.

**Conclusions:**

Bilateral occlusion reveals a fixation-free state of alignment that is different from orthotropia and usually less than the exotropia that occurs spontaneously during binocular viewing. This finding demonstrates that the deviation angle in patients with intermittent exotropia is actively mediated by visual feedback, which the fixating eye is capable of providing alone.

Orthophoria is an ideal but unrealized state of binocular vision. Sensory feedback from both eyes is required to maintain bifoveal fusion.^1^^,^^2^ As soon as one eye is covered, the eyes drift out of alignment. The deviation may be exceedingly small, leading the examiner to conclude erroneously that orthophoria is present. But, if the eyes are tracked with a device of sufficient sensitivity, misalignment will always be detected.^3^

In patients with intermittent exotropia, extraocular muscle tonus exerts a divergent force, referred to as an exophoria.^4^^–^^7^ The exophoria is held in check by an innate drive to fuse the image from each eye. Periodically, fusion breaks down, either spontaneously or from inciting factors such as fatigue, inattention, intoxication, or glare.^8^^–^^11^ One eye then deviates outward to an exotropic position.

In a recent study, we captured eye movement recordings of spontaneous episodes of intermittent exotropia.^12^ The eye movements were indistinguishable from those induced by covering one eye. In both situations, a divergent muscle imbalance was unleashed, causing one eye to become exotropic. In this present study, we have extended these observations by inquiring what happens when both eyes are covered. If disruption of fusion simply released a divergent muscle tonus, one would expect little difference in the magnitude of the exotropia induced by unilateral versus bilateral occlusion. Here, we report that bilateral occlusion usually results in a smaller exotropia than monocular occlusion. This unexpected finding provides new insight into the neural mechanisms underlying intermittent exotropia.

## Methods

### Study Population

For this study, 18 subjects (11 females, 7 males) with typical findings of intermittent exotropia volunteered to participate.^13^^–^^15^ They had a median age of 20 years (range, 8 to 60). They learned about the study from local ophthalmologists or were recruited from the University of California, San Francisco (UCSF) neuro-ophthalmology/pediatric ophthalmology service. Approval for the study was granted by the institutional review boards at UCSF and at Kaiser Permanente Northern California. Informed consent was obtained from adult subjects; minors provided their assent and a parent gave informed consent.

Each subject underwent a complete ophthalmological examination. The eligibility exam included measurement of the best-corrected visual acuity in each eye and assessment of pupils, ocular movements, alignment, and stereopsis (Randot circles and stereo butterfly tests), followed by slit-lamp exam, cycloplegic refraction, and fundus exam. Inclusion criteria were (1) intermittent exotropia since early childhood, (2) 20/20 Snellen acuity in each eye with refractive correction, (3) no eye disease except strabismus, (4) no pathological nystagmus, (5) absence of diplopia, (6) normal stereopsis (at least 60 arcsec), and (7) no history of strabismus surgery. If the subject was eligible and agreed to participate, laboratory testing was scheduled at a later date.

Testing was performed without refractive correction. Subjects with more than 4 diopters of myopia, hyperopia, or astigmatism were excluded.

Testing was limited to subjects with well-controlled intermittent exotropia. The revised Newcastle Control Score was used to evaluate the severity of exotropia.^16^ Only subjects with a score of 3 or less on a scale of 0 to 9 were recruited. Such individuals manifest exotropia infrequently and immediately realign their eyes after dissociation, either spontaneously, with a blink, or with a refixation eye movement.

### Eye Movement Recordings

Each subject was seated comfortably in a chair with the head in an adjustable chin/forehead rest facing a translucent tangent screen at a distance of 57 cm. The screen subtended ±50° horizontally and vertically. A target, consisting of a spot of light 0.5° in diameter, was rear-projected onto the center of the tangent screen with a digital light processing (DLP) projector or a laser. The subject's task was simply to fixate the target. Two video-based eye trackers (iView X; SensoMotoric Instruments, Teltow, Germany), sampling at 60 Hz, were employed to monitor independently the position of each eye. The tracker cameras used infrared light provided by an overhead illuminator with an emission peak of 940 nm. The wavelength emitted by the illuminator was invisible to the subjects. An occluder fastened to a pneumatic piston was positioned to descend rapidly in front of each eye. The occluder was a filter (Schott RG9; Edmund Optics, Barrington, NJ, USA) that passed only infrared light, obscuring the subject's vision and hence the view of the target spot but allowing eye movements to be recorded continuously. The filter shutter blocked entirely the nasal and superior visual field of each eye, and it blocked the central 45° of the temporal and inferior visual field. The background lighting in the room was kept relatively low (10 lux) to enhance the contrast of the target spot. Six subjects were also tested under conditions of either total darkness or normal indoor lighting. The lighting conditions were not found to be an important variable.

The video eye trackers were calibrated to provide a measurement of horizontal and vertical position for each eye. Their accuracy was within ±1°.^17^^,^^18^ Eye, target, and shutter positions, along with pupil diameter, were digitized and acquired for later analysis. Interruptions in eye position tracings caused by blinks were replaced by linear interpolation. Segments marred by excessive blinking or loss of eye tracking were excised. Further details regarding the equipment, experimental design, and tracker resolution have been published.^19^^,^^20^

After calibration of the video trackers, each subject was asked to fixate the central target for a period of 10 seconds. A filter shutter then blocked one eye. After 10 seconds, a second shutter descended over the other eye, blocking all vision except beyond 45° in the far temporal and inferior periphery of the visual field of each eye. After 10 seconds, both shutters opened simultaneously, allowing the subject to regain fusion on the central target. Fifty cycles were tested, alternating initial shutter occlusion between the right eye and the left eye. Subjects were given no instructions regarding where to look during periods of bilateral occlusion. Some subjects became drowsy or fidgety during the experiment, so fewer than 50 cycles could be completed.

Right and left eye horizontal positions were extracted for each trial. The ocular deviation was calculated by subtracting the left eye position from the right eye position. It was plotted as a function of time for 12 seconds, beginning at 2 seconds before the onset of each trial. The mean and standard deviation of the exotropia were calculated over the last portion of each trial, when the ocular deviation had reached a relatively stable value. This time segment was usually 6 seconds but was tailored slightly for each subject. For any given subject, the identical time epoch was analyzed for right eye occlusion, left eye occlusion, and binocular occlusion.

## Results

**Figure 1.**
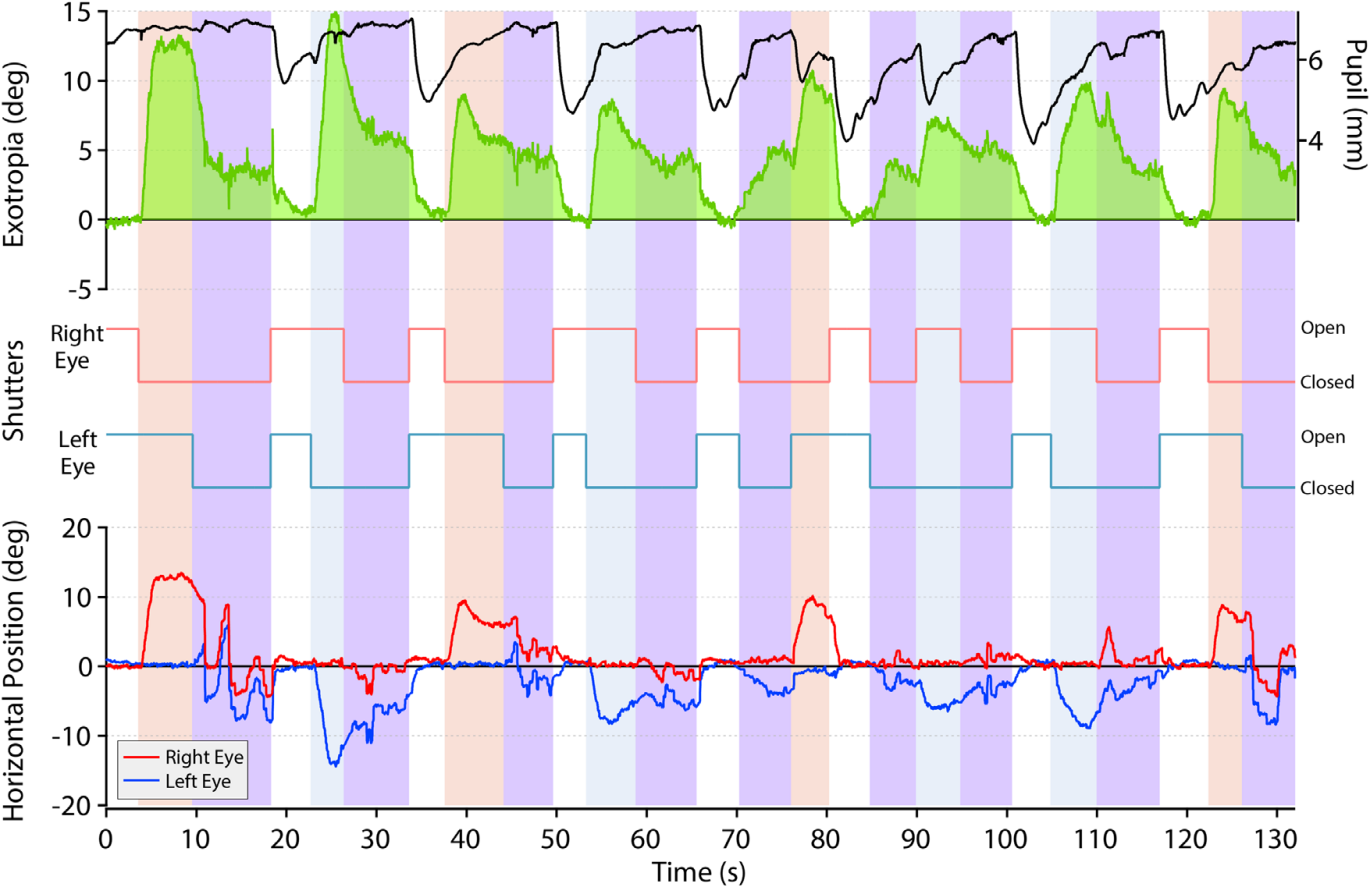
Comparison of exotropia during right eye, left eye, or binocular occlusion. Recordings from a 16-year-old boy (refraction: plano OU) during a 132-second epoch showing the horizontal position of the right eye (*red*) and left eye (*blue*). A 0.5° fixation target was present on a tangent screen at a distance of 57 cm. An occluder located in front of each eye was either open or closed. When one eye was occluded, it became exodeviated while the other eye maintained target fixation. When both eyes were occluded, small saccades were made along with drifts in eye position. The exodeviation became markedly reduced. When neither eye was occluded, fusion was restored. The *green trace* shows changes in the exotropia angle during epochs of binocular viewing, right eye occlusion (*pink shading*), left eye occlusion (*blue shading*), and bilateral occlusion (*purple shading*). Top trace (*black*) shows the pupil diameter.

Figure 1 shows an example of an eye movement recording made in a 16-year-old boy, referred by an ophthalmologist, with a well-controlled intermittent exotropia measured by cover–uncover test at 20 to 25 prism-diopters. The parents reported observing occasionally a spontaneous left eye deviation. To show his eye positions under different viewing conditions, the shutters were opened or closed at variable time intervals (Supplementary Video). Closure of either shutter induced an exodeviation of the occluded eye.^21^ When the shutter was raised, fusion was regained rapidly. When one shutter was closed and subsequently the second shutter was closed, the result was unanticipated: the eyes became less exodeviated. Pupil diameter was smallest when both shutters were open because light exposure was maximal and the eyes were converged. It was largest when both shutters were closed because light exposure was minimal.

**Figure 2.**
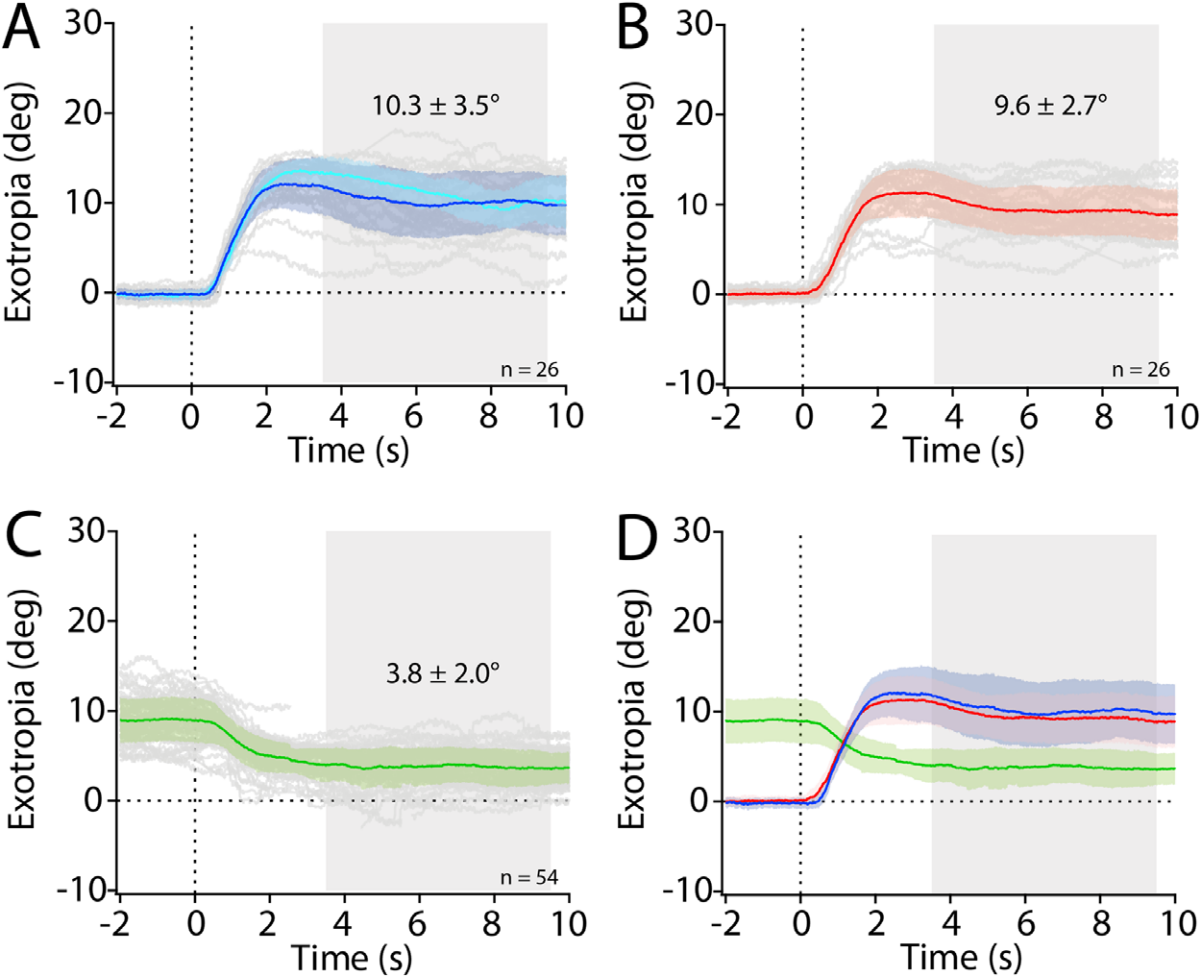
Bilateral occlusion reduces exotropia. Ten-second trials from the subject in Figure 1 showing mean exotropia (*solid line*) and standard deviation (*shading*) during (**A**) left eye occlusion, (**B**) right eye occlusion, and (**C**) bilateral occlusion. Shutter descent occurred at time = 0 seconds. *Gray shading* denotes the 6-second interval when the exotropia ± standard deviation was calculated. In **A**, the *light blue trace* represents mean deviation during eight episodes of spontaneous left exotropia. (**D**) Overlay showing mean exotropia during right eye occlusion (*red trace*), left eye occlusion (*dark blue trace*), and bilateral occlusion (*green trace*). Bilateral occlusion reduced the mean exotropia that was present following monocular occlusion by 60%.

Figure 2 shows data from this patient's main testing session, consisting of repeated cycles of 10-second periods of binocular viewing, right eye or left eye occlusion, and bilateral occlusion. After one shutter closed, the occluded eye began to drift outward with a latency of about 320 ms. By 2 seconds, the maximum excursion was reached and then lessened slightly. The mean left exotropia was 10.3° ± 3.5° (95% confidence interval [CI], 8.9°–11.8°), and the mean right exotropia was 9.6° ± 2.7° (95% CI, 8.5°–10.7°) (compare Fig. 2A and Fig. 2B). Overlap in the 95% CIs indicates that the left exotropia was not significantly different from the right exotropia, as in most patients with intermittent exotropia.^22^

While this patient was viewing the central target with both eyes, he experienced eight episodes of spontaneous left exotropia. These episodes of spontaneous exotropia were similar to those induced by shutter occlusion (compare the light and dark blue traces in Fig. 2A).^12^ No episodes occurred of spontaneous right exotropia.

The effect of binocular occlusion on this subject's ocular deviation is shown in Figure 2C. Covering the second eye reduced the exotropia by 60%. At the beginning of each monocular occlusion trial, the exotropia measured 0°, and the standard deviation was small because the subject was fused. At the beginning of each binocular occlusion trial, the standard deviation of the exotropia was large, because the position of the deviated eye behind the occluder was variable; compare the standard deviations of the exotropia from −2 to 0 seconds in Figures 2A and 2B with that in Figure 2C. As a result, it was difficult to determine precisely the latency for reduction in the ocular deviation produced by binocular occlusion. We measured 340 ms, close to the value of 320 ms for the change in ocular alignment caused by monocular occlusion. Overlay of the deviation traces (Fig. 2D) shows the left and right exotropia, which are similar, and the reduction in exotropia caused by bilateral occlusion. The mean exotropia during monocular occlusion was 10.0° ± 3.1° (95% CI, 9.1°–10.8°). The mean exotropia during binocular occlusion was 3.8° ± 2.0° (95% CI, 3.2°–4.4°). The CIs do not overlap, signifying a reduction in exotropia.

**Figure 3.**
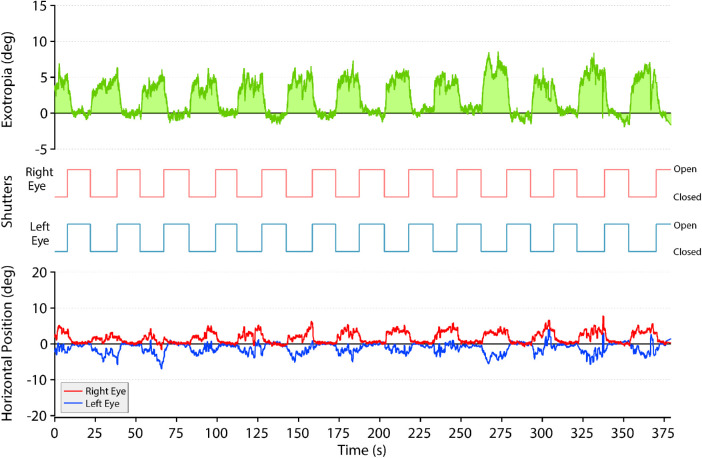
Simultaneous, bilateral shutter closure. Repeated trials from the subject in Figure 1 showing the effect of opening and closing both shutters together. After both shutters closed, both eyes moved outward. This divergence was accompanied by small saccades and conjugate drifts. When both shutters opened, the subject quickly regained fusion. Frequent shifts in eye positions during bilateral occlusion prove that the subject was not engaged in fixation. *Red*, right eye position; *blue*, left eye position.

Next, we investigated whether the magnitude of exotropia during bilateral occlusion depends on both eyes being deprived simultaneously or one eye being occluded before the other. This was tested by opening and closing both shutters while monitoring eye positions (Fig. 3). As soon as both shutters closed, the eyes began to diverge from a fused position, as expected. The latency was 340 ms. This matched the latency recorded for reduction of exotropia from closure of a second shutter (Fig. 2C). After both shutters closed, the eyes diverged fairly symmetrically on each trial (Fig. 3, bottom trace). This observation suggests that the subject did not maintain fixation with one eye on a remembered or imagined central target while the other eye became exotropic. Instead, both eyes participated in the exotropic movement. When both eyes were covered, the subject also made numerous small saccades interspersed with conjugate drifts in eye position.^23^^–^^25^ These eye movements indicate that the subject was not “cheating” by finding an ersatz target to fixate.

**Figure 4.**
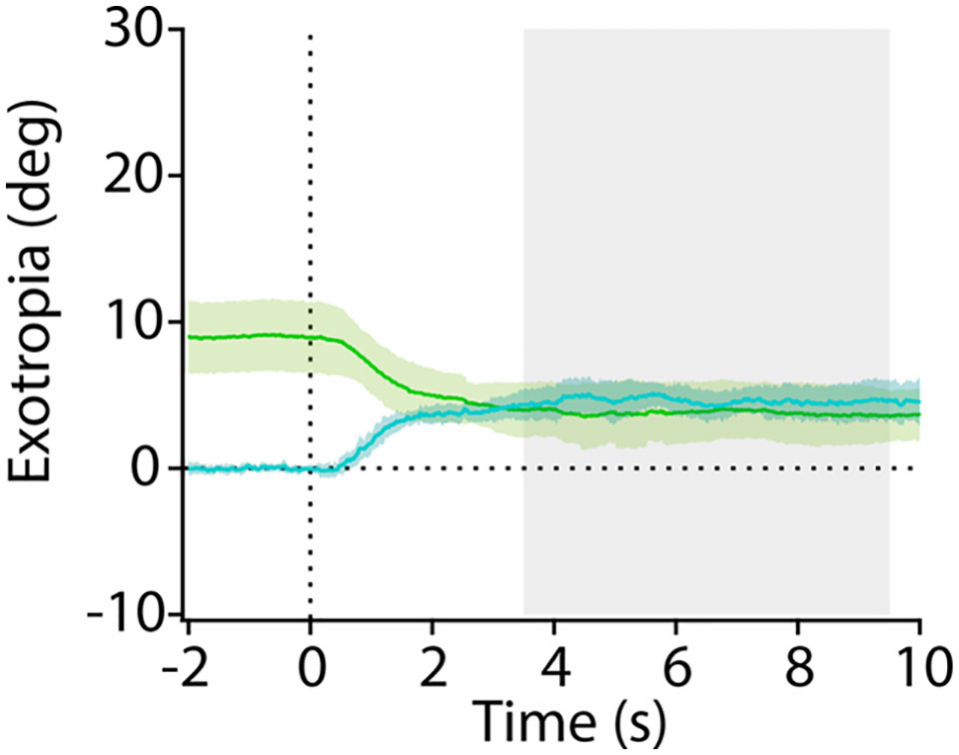
Comparison of exotropia following serial versus simultaneous shutter closure. Traces show mean exotropia (*green*) following staggered closure of the second shutter (Fig. 2C) and mean exotropia (*cyan*) after bilateral, simultaneous shutter closure. Although the relative eye positions were very different at time = 0 seconds when the shutter states changed, the exotropic deviations ultimately attained the same mean value.

After simultaneous closure of both shutters the mean exotropia was 4.6° ± 1.1° (95% CI, 3.9°–5.3°) (Fig. 4). It was not significantly different from the mean exotropia (Fig. 2C) of 3.8° ± 2.0° (95% CI, 3.2°–4.4°) after staggered closure of the shutters.

**Figure 5.**
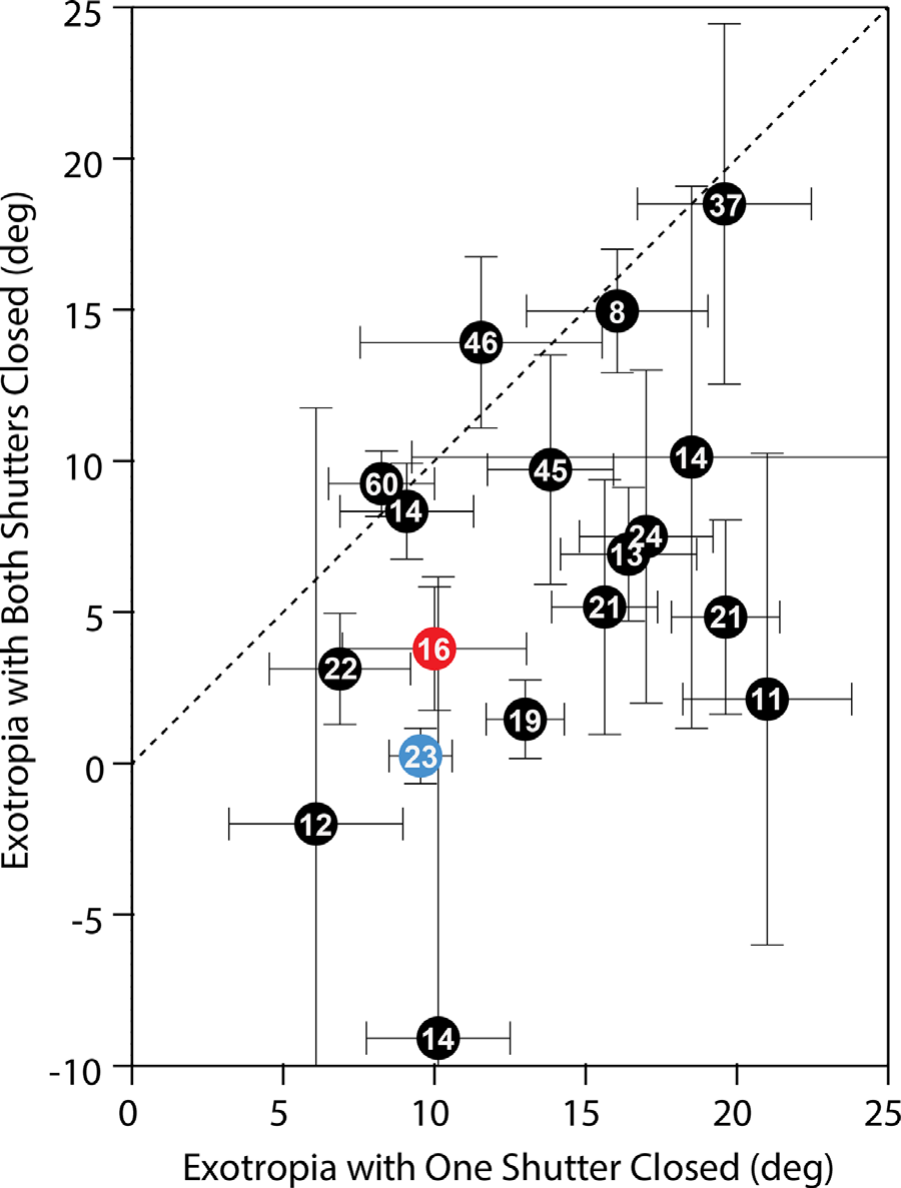
Population data showing reduction of exotropia from bilateral occlusion. Mean exotropia is plotted during closure of one shutter versus both shutters for each subject. Error bars represent 1 SD. Most points fall below the dashed line, signifying a reduction in exotropia from bilateral occlusion. In two subjects, the mean deviation became esotropic. The *red dot* corresponds to the subject illustrated in Figures 1 to 4; the *blue dot* corresponds to the subject in Figure 6. Numbers indicate subject age in years.

Figure 5 compares the magnitude of the ocular deviation under conditions of monocular versus binocular occlusion for all 18 subjects with intermittent exotropia. The impact of bilateral occlusion was heterogeneous. A third of the subjects (7/18) showed little or no effect, judged by the fact that the standard deviation of their exotropia crossed the unity line (Supplementary Fig. S1). These subjects did not differ obviously in their clinical features from the rest of the cohort, except for an older median age (37 years). For the whole population, the mean exotropia with one shutter closed was 13.5° ± 4.7°. The mean exotropia with both shutters closed was 6.0° ± 6.5°, corresponding to a 56% reduction in deviation. The difference in mean exotropia under the two conditions was significant (paired *t*-test, *P* < 0.001).

**Figure 6.**
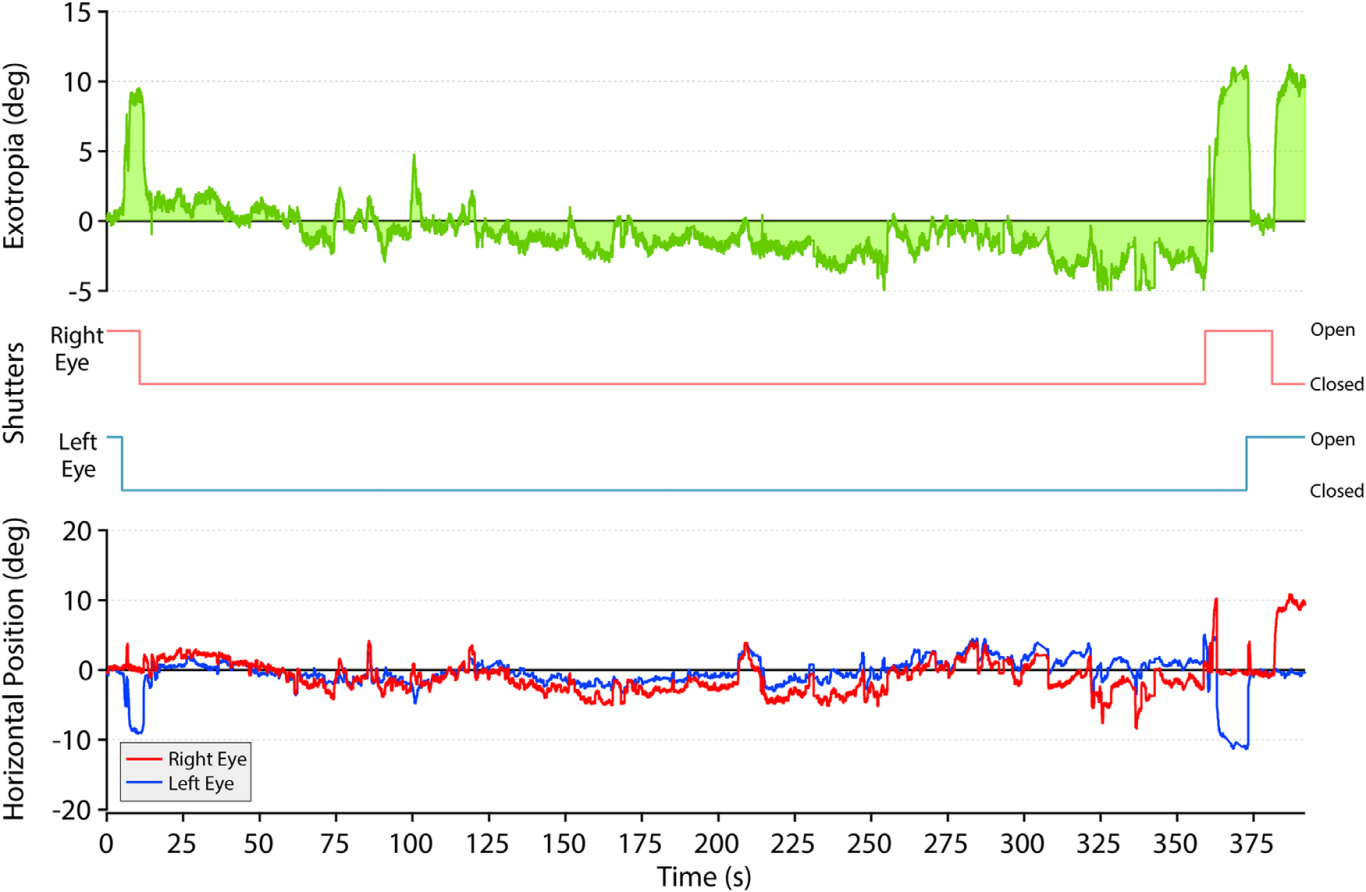
Sustained exotropia reduction during prolonged bilateral visual occlusion. At time = 0 seconds, both shutters are open and the subject is fused. Closure of the left shutter evoked a left exotropia of 10°. Subsequently, closure of the right shutter reduced the exotropia to just a few degrees. While the patient moved her eyes behind the closed shutters, the exotropia gradually diminished and became replaced by an esotropia. After 6 minutes, the right shutter was opened, causing the left exotropia to reappear. The left shutter then opened, and the subject regained fusion. Finally, the right shutter closed to produce a right exotropia. *Red*, right eye position; *blue*, left eye position.

In some subjects, additional testing was performed to determine if the reduction in exotropia persists when bilateral deprivation is maintained. After regular cycles of 10-second occlusion trials were completed, subjects were informed that both shutters would remain closed for a long period. Covering both eyes tended to induce somnolence, causing bilateral ptosis and loss of eye position tracking. However, usable data were collected in five subjects who showed a reduction of exotropia with bilateral occlusion. The reduction of exotropia lasted for the entire period of extended bilateral visual deprivation. A typical set of eye position traces is shown in Figure 6. This 23-year-old woman had a 10° exotropia. It was reduced by bilateral shutter closure, and, in fact, she eventually developed an esotropia. At the end of 6 minutes, one shutter was opened and the 10° exotropia immediately re-emerged. In no subject did bilateral occlusion produce a reduction of exotropia that was sustained beyond the period of bilateral occlusion.

## Discussion

The main finding in this study is that bilateral occlusion reduces the ocular deviation in most patients with intermittent exotropia. When this effect was discovered, our first thought was that a stray reflection or the subject's peripheral vision might be providing a means for attempted fusion. To eliminate these potential sources of artifact, we tested four subjects in total darkness after testing was completed in dim room lighting. The DLP projector cast a glow on the tangent screen, so a laser was substituted to provide a fixation target. Under these conditions, subjects reported that they could detect nothing when the shutters were down. The reduction in exotropia angle still occurred, indicating that subjects were not exploiting a visual stimulus to reduce their misalignment. Further evidence that subjects were not using a substitute target came from the fact that their eyes became engaged in searching behavior as soon as both shutters descended (Fig. 3).

In two subjects, testing was repeated with normal room lighting, after first testing in dim room lighting. A similar exotropia reduction occurred in both subjects. Even under conditions of regular indoor room lighting (500 lux), the infrared shutters create a sensation of relative darkness because they block light, except from the extreme inferior and temporal peripheral visual fields. We attempted to tease apart the effects of form versus light deprivation by conducting the experiment with filters made of ground glass, similar to the frosted translucent occluders used for cover–uncover testing.^26^ Unfortunately, video eye tracking was not possible through ground glass filters. We have no data, therefore, addressing whether obscuration of vision in each eye is sufficient to reduce exotropia or whether darkness is required as well. This issue could be settled by using a different method to record eye positions, such as magnetic search coils.

The resting position of vergence in the absence of vision has been measured by placing normal subjects in the dark. Without fusional or accommodative stimuli, the typical subject converges to a distance of about one meter.^27^^–^^30^ No prior study has tested the behavior of strabismic subjects deprived of visual input. We measured a nearly 60% mean reduction in exotropia magnitude. If this result had been measured in subjects fixating at distance, it might be ascribed to the powerful impetus for convergence driven by bilateral occlusion. However, our subjects were fixating at 57 cm prior to occlusion, so, if anything, bilateral occlusion would have been expected to relax vergence tone. We tested three subjects by presenting a fixation target at both near (57 cm) and distance (300 cm). Bilateral occlusion caused the same reduction of exotropia at near and distance, indicating that the initial vergence angle made no difference.

It is possible that bilateral occlusion reduced exotropia by somehow driving accommodation. We had no means to measure accommodation directly but were able to continuously monitor pupil diameter. Interpretation of pupil data, however, was confounded by changes in light exposure caused by the occluders. When one shutter closed, the pupils dilated (Fig. 1). Subjects remained focused on the fixation target, so accommodation presumably did not change much.^31^ Mydriasis occurred because light exposure was cut by 50% and one eye diverged. When the second shutter closed, the pupils dilated more, but the effect was small because the additional reduction in light was partially offset by convergence. When both shutters opened, the pupils constricted briskly because light exposure increased and the eyes converged further to recover fusion.

The subjects’ accommodative state while both eyes were occluded was unknown, but it seems unlikely that a further increase in accommodation was the primary mechanism driving the reduction in exotropia, for three reasons. First, before descent of the second shutter, subjects were already viewing a target at 57 cm, stimulating an average of 1.75 diopters of accommodation. Second, after descent of the second shutter, no fixation target was present to promote accommodation. Third, accommodation and vergence become partially dissociated in the dark.^32^ This uncoupling suggests that, under our testing conditions, accommodation should not be a powerful driver of convergence. Nonetheless, we note that three out of seven subjects who showed no effect were older (ages 60, 46, and 37), raising the possibility that accommodative ability might be important. However, four out of the seven subjects who showed no effect were young (ages 8, 12, 14, and 14).

It has been suggested on the basis of observations in deceased subjects^33^ and patients administered curare^34^ that the absolute position of rest for the eyes is divergent. This bias is proposed to be enhanced in intermittent exotropia due to abnormal properties of the extraocular muscles.^35^^,^^36^ Structural changes in myofibrils and connective tissue are thought to alter muscle tonus, leading to an excess of divergent forces that are normally held in check by the countervailing drive to maintain fusion. A major problem with such mechanical theories is that it is difficult to determine whether the putative anatomical changes are a cause or a result of strabismus. In either case, if occlusion breaks fusion and allows the eye to rotate outward due to muscle tonus imbalance, much like the release of a compressed spring, then it should make no difference if one eye is occluded or both eyes are occluded. Our results strongly argue against the concept that exotropia is due to an intrinsic abnormality of the extraocular muscles that pulls the eyes outward.

Our experiments have revealed in intermittent exotropia a major, unsuspected difference between abolishing fusion and abolishing fixation. The ocular posture assumed under the latter condition was termed the “static position” by Lancaster,^37^ and it was referred to as the “fixation-free” position by Spielmann.^26^ She made it easy for the clinician to observe the position of the eyes under fixation-free conditions by introducing the use of translucent occluders. Remarkably, she made the anecdotal comment that “not infrequently” patients with exotropia return to orthoposition under bilateral translucent covers.^26^ This prescient observation suggests that simply preventing fixation is enough to reduce exotropia, and light attenuation by the filters is not required. It remains to be determined whether it is the loss of fixation per se or elimination of all high grade form vision that produces this effect.

A common misconception is that, when a patient with intermittent exotropia loses fusion, the deviating eye becomes suppressed completely. Dichoptic visual field testing has shown that suppression is limited to the peripheral temporal retina in each eye, and remarkably, both foveae continue to contribute to perception.^38^^–^^42^ Subjects with intermittent exotropia flip back and forth between two active, binocular sensory states: fused and deviated. It makes no difference in the magnitude of the ocular deviation whether the subject views with both eyes or only one eye. This point is illustrated in Figure 2A and is supported by comparison of spontaneous exotropia with occluder-induced exotropia.^12^

Occlusion of both eyes gives rise to a third sensory state. The resulting deviation is generally smaller than the exotropia that is present during binocular or monocular viewing. This difference provides a clue to the pathogenesis of intermittent exotropia. It means that the exotropia that appears after fusion loss is not simply a default state imposed by abnormal muscles that produce excessive divergence tonus. Rather, the deviation angle in intermittent exotropia is under active control provided by visual feedback. One eye is sufficient to provide this feedback, given that covering the other eye makes no difference. But, when both eyes are covered, abolishing all visual feedback, they usually drift to a novel position.

## Supplementary Material

Supplement 1

Supplement 2

## Supplementary Material

**Supplementary Video.** Sixteen-year-old-boy with an intermittent left exotropia fixating on a target at 57 cm while the right eye, left eye, or both eyes are occluded with a filter that blocks his vision but allows the position of each eye to be tracked by a camera using infrared light. Data from this subject are shown in Figures 1 to 4. Horizontal positions are plotted for the right eye (*red*) and the left eye (*blue*), with positive values equal to right gaze. The main finding is that monocular occlusion results in a much larger exotropia than binocular occlusion.
