# Supplementary material for: Bilateral Occlusion Reduces the Ocular Deviation in Intermittent Exotropia
Source: Invest Ophthalmol Vis Sci. 2021 Jan 5;62(1):6. doi: 10.1167/iovs.62.1.6 (PMC7794258; doi:10.1167/iovs.62.1.6)
Supplement: Supplement 1 [file iovs-62-1-6_s001.pdf]

Supplementary Figure 1

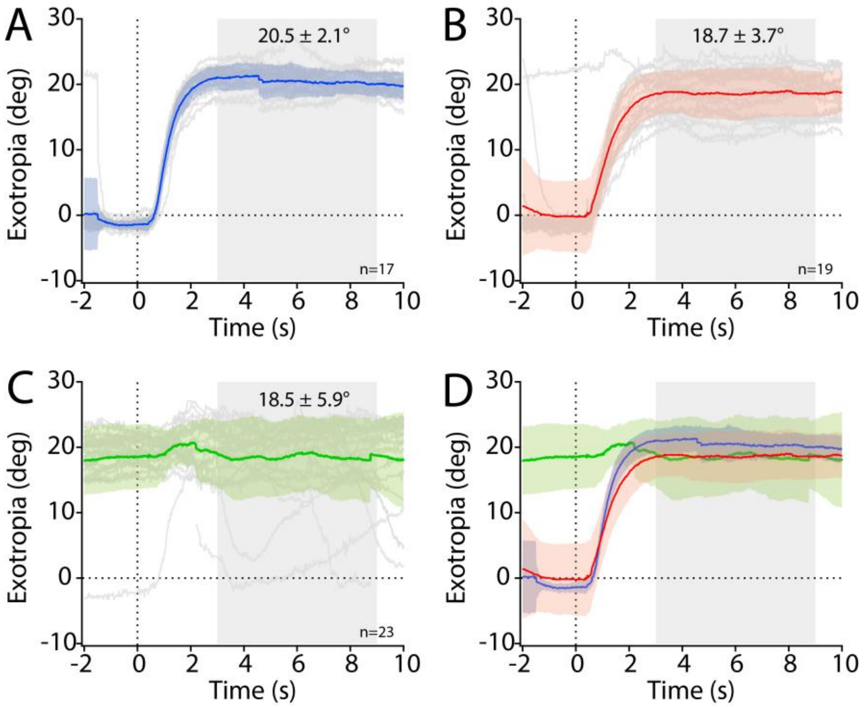

Supplementary Figure 1) Eye position traces showing no effect of bilateral occlusion in a 37-year-old subject. **A)** left eye occlusion. **B)** right eye occlusion. **C)** bilateral occlusion. **D)** Comparison of means of all 3 conditions. Shutter descent occurred at time = 0 seconds. Gray shading indicates the 6 second interval when the exotropia ± standard deviation was calculated. In this subject, bilateral occlusion did not reduce the mean exotropia present during occlusion of either the right eye or the left eye.
